# Ferroptosis in diabetes-associated cognitive dysfunction: mechanisms and therapeutic potential

**DOI:** 10.3389/fphar.2026.1756129

**Published:** 2026-03-10

**Authors:** Hang Liu, Siqi Qian, Shuzhen Liu, Wenbiao Xiao, Yijie You, Yi Zeng

**Affiliations:** 1 Department of Geriatrics, The Second Xiangya Hospital of Central South University, Changsha, China; 2 Department of Radiology, The Second Xiangya Hospital of Central South University, Changsha, China; 3 Xiangya School of Medicine, Central South University, Changsha, China

**Keywords:** diabetic-associated cognitive dysfunction, ferroptosis, iron metabolism, lipid peroxidation, pathogenesis, therapeutic target

## Abstract

Diabetic-Associated Cognitive Dysfunction (DACD) is a major central nervous system complication of diabetes. Its pathogenesis involves dysfunction of the neurovascular unit, oxidative stress, and chronic neuroinflammation. Ferroptosis, an iron-dependent form of regulated cell death, is strongly implicated in DACD progression. This review synthesizes its core mechanisms, focusing on three key areas: metabolic iron overload, aberrant lipid peroxidation, and disruptions in key amino acid pathways. These processes collectively drive ferroptotic damage in vulnerable neurons of the hippocampus and cortex, ultimately leading to cognitive decline. Furthermore, this work highlights the translational potential of targeting ferroptosis using specific inhibitors (e.g., Ferrostatin-1, Deferoxamine, Erythropoietin) for DACD treatment, offering novel strategic insights for intervention. Future clinical investigations are essential to validate the efficacy of these therapeutic targets in diabetic patient populations.

## 1 Introduction

According to the 11th edition of the International Diabetes Federation (IDF) Diabetes Atlas, released in 2025, the global number of adults aged 20–79 years living with diabetes was estimated to be 589 million in 2024, corresponding to a prevalence of 11.11%. Driven by factors such as dietary changes and increasing obesity, the global diabetes epidemic continues to rise, with projections indicating an increase to 853 million people (12.96% prevalence) by 2050 (Genitsaridi et al., 2026). Diabetes mellitus is a metabolic disease characterized by chronic hyperglycemia of diverse etiologies, which induces long-term damage across multiple organ systems.

In a key neuropathological investigation, Reske-Nielsen et al. (1966) identified a distinct histological pattern featuring diffuse degenerative abnormalities in the brain tissue of diabetic patients upon autopsy, a finding which justified the introduction of the concept of “diabetic encephalopathy”. Subsequent epidemiological research has established a robust association between diabetes and dementia, specifically Alzheimer’s disease and vascular dementia, encompassing shared risk factors such as aging, comparable peak ages of onset, and certain shared genetic predispositions. Mijnhout et al. (2006) noted the inadequacy of the term “diabetic encephalopathy” in highlighting cognitive alterations in diabetic patients and consequently introduced the nomenclature “Diabetes-Associated Cognitive Dysfunction” to improve clinical recognition of this condition.

Diabetes-induced damage to the central nervous system (CNS) has emerged as a significant concern. The neurological complications of diabetes are multifaceted, encompassing cerebrovascular disease and acute metabolic crises such as ketoacidotic, hyperosmolar hyperglycemic, and hypoglycemic coma. Nevertheless, cognitive dysfunction associated with diabetes frequently receives insufficient attention. DACD is defined as a decline in one or more cognitive domains and is recognized as a prevalent complication of both type 1 and type 2 diabetes (Yu et al., 2025). According to the Diagnostic and Statistical Manual of Mental Disorders, Fifth Edition, cognitive function is categorized into the following domains: complex attention, executive function, learning and memory, language, perceptual-motor ability, and social cognition (Sachdev et al., 2014). Cognitive dysfunction is characterized by impairments in one or more cognitive domains and is frequently accompanied by mental, behavioral, and personality abnormalities during the disease course. Diabetes is established as a key modifiable risk factor for dementia, with midlife onset being particularly significant (Livingston et al., 2024). The precise pathogenesis of DACD remains to be fully elucidated. Current research has predominantly focused on the interplay of neurovascular unit dysfunction, blood-brain barrier disruption, oxidative stress, advanced glycation end products, and neuroinflammatory responses in mediating neuronal injury (Gao L. et al., 2025; Yan et al., 2020; Ying et al., 2024).

However, the specific role of ferroptosis in this pathogenic cascade has been largely overlooked. Cell death is a fundamental biological process denoting the irreversible termination of cellular activities in living organisms. Its main classifications include accidental cell death and regulated cell death. Regulated cell death encompasses multiple forms such as apoptosis, autophagic cell death, pyroptosis, necroptosis, and ferroptosis (Denton and Kumar, 2019; Mou et al., 2019; Tong et al., 2022). Ferroptosis, a form of iron-dependent regulated cell death first termed in 2012, is primarily driven by the inactivation of glutathione peroxidase 4 (GPX4), resulting in the accumulation of lipid peroxides (Dixon et al., 2012; Sui et al., 2018). Ferroptosis is driven by dysregulation in three core metabolic pathways: thiol metabolism, lipid peroxidation, and iron metabolism (Dixon et al., 2012). Within this framework, thiols, such as glutathione (GSH), function as crucial intracellular antioxidants essential for maintaining redox balance. Depletion of GSH or disruption of its metabolism, such as reduced GPX4 activity, impairs cellular antioxidant capacity. This compromises the clearance of reactive oxygen species (ROS) generated during lipid peroxidation, thereby facilitating ferroptosis execution. Ferroptosis is driven by pervasive lipid peroxidation. Its mechanism involves the Fenton reaction, in which iron ions catalyze the formation of hydroxyl radicals (OH). These radicals initiate and propagate the peroxidation of polyunsaturated fatty acids (PUFAs) in cellular membranes. The consequent accumulation of peroxidized lipids disrupts vital membrane functions, resulting in content leakage and cell death. The intracellular accumulation of iron, which can result from upregulated uptake via the transferrin receptor 1 (TfR1) or impaired storage within ferritin, is a key driver of ferroptosis. The accumulated iron subsequently contributes to lipid peroxidation through two principal mechanisms: by participating in the Fenton reaction to generate ROS, and by serving as an essential cofactor for lipoxygenases (LOXs), thereby directly catalyzing membrane lipid peroxidation. Thiol metabolism, lipid peroxidation, and iron metabolism constitute the fundamental regulatory axis of ferroptosis, whose dysregulation is a pivotal trigger for its initiation.

**FIGURE 1:**
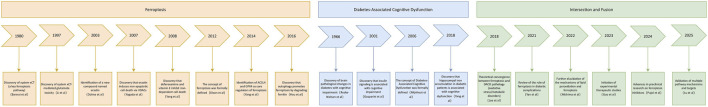
Timeline of research development on ferroptosis and diabetes-associated cognitive dysfunction.

It is increasingly recognized that ferroptosis, a regulated form of cell death, plays a critical role in diabetes and its complications, with its underlying mechanisms being continuously uncovered by recent studies. The established strong link between ferroptosis and the pathogenesis and progression of DACD underscores the necessity for continued investigation in this research area (Liu et al., 2024) (Figure 1). Despite the existing body of reviews describing ferroptosis in the context of diabetes pathophysiology and complications (Liu et al., 2024; Yan et al., 2021), there is a notable gap in the literature regarding its specific contribution to DACD. This review addresses this specific gap by providing a systematic synthesis of the core molecular mechanisms of ferroptosis within DACD. It details how three interlinked pathological events—iron overload due to metabolic dysregulation, aberrant lipid peroxidation, and disruption of key amino acid pathways—are activated in the diabetic context to drive neuronal loss. By elucidating how these systemic metabolic disturbances converge to induce selective ferroptotic damage in vulnerable hippocampal and cortical neurons, the work establishes a direct mechanistic link between general diabetic complications and cognitive decline, thereby bridging the related research fields. Furthermore, the review evaluates the therapeutic potential of targeting this pathway with specific ferroptosis inhibitors, connecting the mechanistic exploration directly to novel intervention strategies for DACD and outlining a clear rationale for future clinical validation.

## 2 The mechanism of ferroptosis in diabetes-associated cognitive dysfunction

Iron, an essential trace element, crosses the blood-brain barrier via a complex metabolic regulatory network and participates in diverse critical biological processes within the central nervous system, including oxygen transport, energy metabolism, and the synthesis and catabolism of myelin and neurotransmitters (Gao Q. et al., 2025). Growing evidence has established that ferroptosis, a novel modality of regulated cell death, is implicated in a wide range of biological processes via a sophisticated regulatory network encompassing various signaling molecules and metabolic pathways (Gao et al., 2015; Ursini and Maiorino., 2020). The following discussion focuses on elucidating the roles of iron, lipid, and amino acid metabolism in the pathogenesis of ferroptosis-related DACD (Table 1).

**TABLE 1:** Summary of the ferroptosis core pathway and its dysregulation in Diabetes-associated cognitive dysfunction.

Core pathway	Key regulatory molecules	Dysregulation	Pro-ferroptotic consequence
Iron metabolism	TfR1, FPN1, Ferritin (FTH1/FTL), Hepcidin​	Iron overload; Increased TfR1 expression; Decreased FPN1 and Ferritin expression; Elevated cortical/hippocampal iron deposition	Increased labile iron pool fuels the Fenton reaction, generating ROS for lipid peroxidation
Lipid metabolism	ACSL4, LPCAT3, PUFAs (e.g., AA, AdA), PE	Increased incorporation of PUFAs into membrane phospholipids (e.g., PE)	Increases the abundance of peroxidation-susceptible phospholipids, providing substrate for lipid peroxidation
Amino acid metabolism			
Xc-/GSH/GPX4 Pathway	GSH, GPX4	Inhibition of System Xc- activity; Depletion of GSH; Inactivation of GPX4	Loss of the primary enzymatic defense against phospholipid hydroperoxides, leading to their accumulation
FSP1/CoQ/DHODH Pathway	FSP1, CoQ, DHODH	Impaired FSP1 function; Depletion of the cellular CoQH2 pool; Pharmacological inhibition of DHODH	Compromises a non-GSH dependent, membrane-associated antioxidant defense system
GCH1/BH4 Pathway	GCH1, BH4	Inhibition or reduced activity of the pathway (e.g., potentially via dysregulation of upstream modulators like Spy1)	Compromises endogenous defense by reducing BH4 synthesis and increasing membrane peroxidation susceptibility, thereby promoting ferroptosis
NRF2/SLC7A11/GPX4 Pathway	NRF2, SLC7A11, GPX4	Inhibition of NRF2 signaling (e.g., by ML385), leading to failed transcriptional activation of SLC7A11 and GPX4	Impairs cystine uptake and lipid peroxide reduction, thereby crippling glutathione-dependent antioxidant defense and leading to unchecked lipid peroxidation and ferroptosis execution
AMPK pathway	AMPK, ACC	Inflammatory stimuli and elevated ROS modulating the pathway	Increases neuronal susceptibility to ferroptosis, contributing to damage in critical brain regions

### 2.1 Iron metabolism

Dietary iron constitutes the principal exogenous supply of this essential element for humans. The metabolism of dietary iron initiates with its absorption into the bloodstream primarily in the duodenum and upper jejunum. This process occurs via two distinct pathways: heme iron, derived from animal sources, is internalized by enterocytes through the heme carrier protein 1 (HCP1), whereas non-heme iron, present in both plant and animal foods, must first be reduced from Fe^3+^ to Fe^2+^ form by ferrireductases such as duodenal cytochrome b (Dcytb) before it can be imported into the cell via the divalent metal transporter 1 (DMT1). Within intestinal enterocytes, dietary iron is exported into the systemic circulation via the iron exporter ferroportin-1 (FPN1) located on the basolateral membrane; surplus iron that is not immediately exported is stored within the cytosolic protein ferritin. The liver-derived hormone hepcidin serves as the principal regulator of systemic iron homeostasis by binding to FPN1 on the surface of enterocytes and other cell types, thereby inducing its internalization and degradation and ultimately inhibiting iron entry into the bloodstream (Tayal et al., 2025). The majority of absorbed iron is delivered to the bone marrow for heme synthesis in erythroid cells. Iron released into the circulation, whether from dietary absorption or recycling of senescent red blood cells, is oxidized from Fe^2+^ to Fe^3+^ state by the ferroxidase ceruloplasmin. This oxidized iron then binds to transferrin (TF) in the plasma, forming holotransferrin, which is subsequently taken up by cells via TfR1-mediated endocytosis (Li J. et al., 2020). Under the acidic conditions within the endosome, iron is released from transferrin. Fe^3+^ is subsequently reduced to Fe^2+^ by the metalloreductase six-transmembrane epithelial antigen of the prostate 3 (STEAP3), followed by its transport into the cytosol via DMT1 to join the labile iron pool (LIP) or be stored in ferritin (Yan et al., 2021; Zhou et al., 2020). The iron-sequestering protein ferritin is a multimeric complex composed of both ferritin light chain (FTL) and heavy chain (FTH1) subunits. Cellular iron export is facilitated by FPN1, the sole known iron exporter, which is encoded by Solute Carrier Family 40 Member 1(SLC40A1) gene. The efflux of Fe^2+^ via FPN1 is coupled to its oxidation to Fe^3+^ state, a reaction essential for iron binding to transferrin in the circulation (Tang et al., 2021; Xie et al., 2016). Brain iron metabolism is a tightly regulated process initiated by the transport of iron across the blood-brain barrier (BBB). The BBB, which is primarily composed of brain microvascular endothelial cells (BMVECs) in close association with astrocytic endfeet, serves as the principal gateway that stringently controls the entry of iron into the brain parenchyma (Dong et al., 2025). Transferrin-bound iron (TF-Fe) gains entry into BMVECs by binding to TfR1 on the plasma membrane, initiating receptor-mediated endocytosis and subsequent encapsulation within endosomes. Within the acidic endosomal compartment, Fe^3+^ dissociates from transferrin and is reduced to its ferrous form Fe^2+^ prior to its transport into the cytosol via DMT1. The export of cytosolic iron into the brain interstitial fluid is mediated by FPN1 located on the basolateral membrane of cells. Subsequently, Fe^2+^ is oxidized to Fe^3+^ by the ferroxidase ceruloplasmin (CP), which is primarily produced by astrocytes in the brain, enabling its reloading onto transferrin to complete the iron recycling process (Dong et al., 2025; Kulaszyńska et al., 2024). Following its entry into the brain interstitial fluid, TF-Fe enters neurons and various glial cells via endocytosis, mediated chiefly by the TfR1 or alternatively by the DMT1, thus supporting their distinct metabolic demands (Kulaszyńska et al., 2024; Qian and Ke, 2019).

This process is part of a broader, strictly orchestrated system where the absorption, distribution, and utilization of iron within the central nervous system are governed by a sophisticated, multicellular and multilevel regulatory framework. Consequently, iron homeostasis within each neural cell is maintained by a dynamic equilibrium between iron import and export mechanisms. The iron handled through these processes is indispensable for the function of enzymes critical to pathways such as phospholipid peroxidation and ROS metabolism. A disruption in the homeostasis of iron metabolism, manifesting as an imbalance among its import, storage, and export, can lead to intracellular iron overload. The accumulated iron subsequently promotes lipid peroxidation primarily via the Fenton and Haber-Weiss reactions, thereby triggering the iron-dependent cell death process known as ferroptosis (Yan et al., 2021; Zhou et al., 2020; Xie et al., 2016).

**FIGURE 2:**
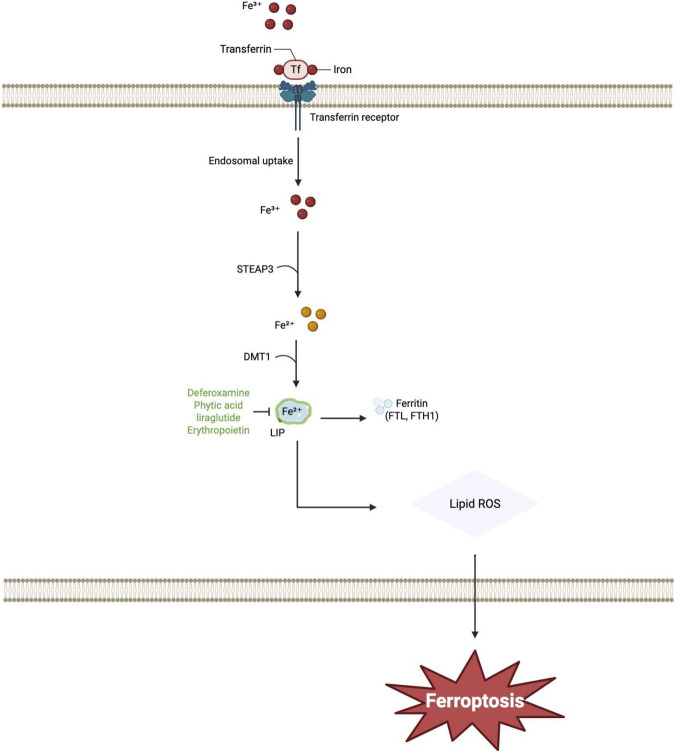
The main molecular mechanisms of ferroptosis-related diabetes-associated cognitive dysfunction include iron metabolism. Extracellular Fe^3+^ binds to transferrin and enters the cell via transferrin receptor-mediated endocytosis. Within the endosome, Fe^3+^ is reduced to Fe^2+^ by STEAP3, then transported into the cytosol via DMT1 to form the LIP. Iron from the LIP can either be stored in ferritin or drive the production of Lipid ROS, ultimately leading to ferroptosis. Abbreviations: DMT1, Divalent Metal Transporter 1; LIP, Labile iron pool; ROS, Reactive Oxygen Species; STEAP3, Six-transmembrane epithelial antigen of prostate 3.

Iron overload, resulting from dysregulated iron metabolism, is a well-established initiating factor for ferroptosis (Fang et al., 2023). Consistent with this mechanism, iron deposition in the hippocampal region of patients with type 2 diabetes mellitus (T2DM) is causally linked to cellular damage via oxidative stress, leading to cognitive impairment that is inversely correlated with cognitive performance metrics (Yang et al., 2018). A proposed mechanism involves the Triggering Receptor Expressed on Myeloid cells 1 (TREM1), which induces microglial ferroptosis mediated by the Protein kinase R-like Endoplasmic Reticulum Kinase (PERK) pathway of the endoplasmic reticulum stress (ERS) response (Zhao Q. et al., 2025). The targeted reduction of FPN1 expression has been shown to ameliorate hippocampal iron overload in T2DM rodent models, which in turn improves cognitive impairment (Liu et al., 2020). Complementary investigations indicate that a dysregulation of iron metabolism, characterized by increased TfR1 expression coupled with decreased levels of both FPN1 and FTL, elevates the labile iron pool within astrocytes. This dysregulation promotes astrocytic ferroptosis, a process that subsequently impairs neuronal viability and function, ultimately contributing to the pathogenesis of DACD (Wang et al., 2024). Quantitative susceptibility mapping (QSM) studies have demonstrated that patients with T2DM and cognitive impairment exhibit heightened iron deposition in specific brain regions, including the right caudate nucleus, substantia nigra, and left putamen, compared to their cognitively intact counterparts. This regional iron accumulation is implicated in the pathophysiology of cognitive decline associated with T2DM (Yang et al., 2018). The underlying mechanism may involve disruptions to the corto-striato-thalamo-cortical circuit, particularly affecting the anterior striatal pathway (Sui et al., 2025). Supporting this, a recent prospective cohort study has confirmed that significant iron deposition in the caudate nucleus and putamen of patients with T2DM is significantly associated with diminished cognitive performance, manifesting as slower processing speed, declined memory, and poorer executive function, thereby indicating a negative correlation between the degree of iron accumulation in these nuclei and overall cognitive ability (Cheng et al., 2025). Conversely, QSM-based measurements have revealed localized iron reduction in the temporo-occipital cortex of T2DM patients with mild cognitive impairment, hinting at a potential reorganization of brain iron in the early stages of DACD (Zhao Y. et al., 2025). Converging evidence indicates that dysregulated iron metabolism constitutes a core pathological mechanism in diabetic cognitive dysfunction (Figure 2).

Under diabetic conditions, cerebral iron homeostasis is disrupted, leading to abnormal iron deposition in regions such as the hippocampus, caudate nucleus, putamen, and substantia nigra. The resultant iron overload catalyzes lipid peroxidation via the Fenton reaction, subsequently inducing ferroptosis in critical neural cells, including astrocytes and microglia. This cascade ultimately impairs neuronal survival and function, contributing to cognitive decline. Consequently, therapeutic targeting of the ferroptosis pathway emerges as a promising intervention strategy for DACD.

### 2.2 Lipid metabolism

A comprehensive network of lipid metabolic pathways, including fatty acid synthesis, uptake, β-oxidation, phospholipid synthesis/remodeling, and lipid storage/release, interfaces with the cellular antioxidant defense machinery, thereby influencing the cell’s vulnerability to ferroptosis (Lin et al., 2021). Fatty acid β-oxidation typically functions as a suppressor of ferroptosis by diminishing the intracellular pool of unesterified PUFAs, which are essential substrates for lipid peroxidation. Concurrently, the processes of phospholipid synthesis and remodeling dictate the oxidative propensity of cellular membranes by influencing their composition, thereby directly modulating the cell’s susceptibility to ferroptosis. The pivotal enzymes acyl-CoA synthetase long-chain family member 4 (ACSL4) and lysophosphatidylcholine acyltransferase 3 (LPCAT3) are central to phospholipid esterification and remodeling; genetic or pharmacological inhibition of either enzyme effectively impedes the progression of ferroptosis (Dixon et al., 2015; Huang et al., 2024).

ACSL4 enzymatically activates long-chain PUFAs, such as arachidonic acid (AA) and adrenic acid (AdA), by conjugating them with coenzyme A (CoA) to form PUFA-CoA derivatives (Lee et al., 2023). This activation facilitates the subsequent incorporation of these PUFAs into membrane phospholipids, thereby promoting an increase in membrane-associated, PUFA-containing phospholipid species (Ma et al., 2021). LPCAT3 functions in concert with ACSL4, catalyzing the esterification of lysophospholipids to generate PUFA-containing phospholipids, such as phosphatidylcholine (PC) and phosphatidylethanolamine (PE). (Mishima and Conrad, 2022; Thomas et al., 2018). Notably, PE demonstrates high susceptibility to lipoxygenase-mediated peroxidation, establishing it as a key phospholipid class responsible for inducing cellular ferroptosis (Kagan et al., 2017). The regulation of ferroptosis is influenced by the balance of lipid storage and release. Sequestration of PUFAs within lipid droplets enhances their resistance to peroxidative damage while simultaneously serving as a reservoir for subsequent PUFA utilization in phospholipid biosynthesis (Chen et al., 2021; Danielli et al., 2023). Critically, ferroptosis is driven by lipid peroxidation, a process initiated when ROS attack oxidation-sensitive lipids within cellular membranes (Liang et al., 2022; Von Krusenstiern et al., 2023; Wan et al., 2019). Lipid peroxidation occurs via two distinct mechanisms: enzymatic and non-enzymatic pathways. Critically, the non-enzymatic pathway possesses the capacity to initiate ferroptosis independently of enzymatic activity (Fujii and Imai, 2024). The peroxidation process comprises a chain reaction whereby ROS-initiated hydrogen abstraction from PUFA-phospholipids generates a lipid radical; its subsequent oxygenation yields a peroxyl radical that attacks neighboring phospholipids, culminating in lipid hydroperoxide formation. Endogenous antioxidant systems, including the Xc-/GSH/GPX4 axis, function to scavenge these radicals, thereby quenching the chain reaction and curtailing oxidative damage (Valgimigli L, 2023; Naowarojna et al., 2023; Sun et al., 2025). The peroxidation of membrane phospholipids containing PUFAs compromises membrane integrity, elevates permeability, and generates cytotoxic aldehydes, including 4-hydroxynonenal (HNE) and malondialdehyde (MDA). This cascade leads to the excessive accumulation of lipid hydroperoxides, which ultimately triggers the iron-dependent cell death pathway of ferroptosis (Naowarojna et al., 2023).

The hypothalamus acts as an integrative center for systemic metabolism, modulating lipid levels through multiple pathways. Its lipid metabolism significantly influences feeding behavior and glucose homeostasis. Animal studies confirm that chronic high-fat diet impairs hypothalamic lipid sensing, a dysfunction that can be ameliorated to control feeding behavior by reducing LCFA oxidative metabolism (Pocai et al., 2006), while more recent evidence highlights the critical role of specific neuronal nutrient sensors in this process (Fosch et al., 2023). Elevated levels of free fatty acids (FFAs) have been implicated in the pathogenesis of cognitive impairment. Excessive FFAs can promote the accumulation of amyloid-β oligomers and hyperphosphorylation of tau protein, contributing to cognitive decline. Approximately 25% of the body’s total cholesterol resides in the brain, where it serves as a critical component of neuronal membranes and myelin. The brain synthesizes all cholesterol locally due to the blood-brain barrier, which prevents exchange with peripheral lipoproteins; consequently, cholesterol homeostasis relies on an apolipoprotein E (ApoE)-dependent recycling pathway. ApoE participates critically in neural growth and repair mechanisms. The genetic ablation of ApoE compromises neuronal synaptic integrity and plasticity, consequently inducing lipid peroxidation and cognitive dysfunction. Among its isoforms, ApoEε4 demonstrates the strongest association with DACD. Epidemiological evidence indicates that T2DM patients carrying the ApoEε4 allele face a substantially elevated risk of developing Alzheimer’s disease and mixed dementia, with hazard ratios of 4.58 and 3.89, respectively (Irie et al., 2008). A meta-analysis further quantifies that, within the high-risk population of ε4 carriers, the presence of T2DM confers an additional 35% increased risk of incident all-cause dementia (Relative Risk = 1.35) (Li J. et al., 2020). ROS driven by ferroptosis induce significant cellular oxidative stress, which contributes to the progression of late-stage complications in T2DM mediated through inflammatory signaling pathways (Niazpour and Meshkani, 2025).

**FIGURE 3:**
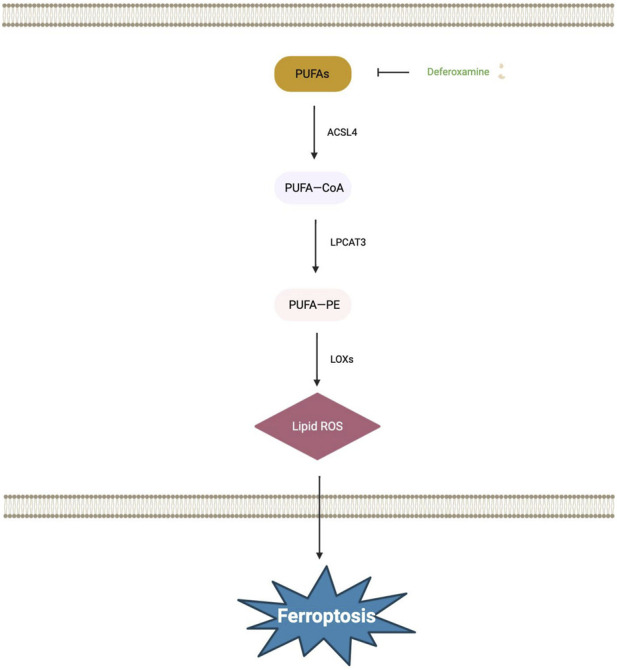
The main molecular mechanisms of ferroptosis-related diabetes-associated cognitive dysfunction include lipid metabolism. PUFAs are first activated to PUFA-COA under the catalysis of ACSL4. Subsequently, they are esterified into membrane phospholipids by the action of LPCAT3 to form PUFA-PE. This specific phospholipid substrate then undergoes peroxidation catalyzed by LOXS, generating the critical toxic end product-Lipid ROS. The uncontrolled accumulation of Lipid ROS ultimately triggers ferroptosis in cells. Abbreviations: ACSL4, Acyl-CoA synthetase long-chain family member 4; LOXS, Lipoxygenases; LPCAT3, Lysophosphatidylcholine Acyltransferase 3; PUFAs, Polyunsaturated Fatty Acids; PUFA-COA, Polyunsaturated Fatty Acyl-CoA; PUFA-PE, Polyunsaturated Fatty Acyl-Phosphatidylethanolamine.

Studies utilizing mouse models of T2DM-associated cognitive dysfunction and high glucose-stimulated PC12 cell models have established a link between DACD pathogenesis and ferroptosis mediated by GPX4 suppression. Evidence confirms that erythropoietin alleviates neuronal ferroptosis by inhibiting lipid peroxidation, thereby mitigating DACD symptoms (Guo et al., 2023). The suppression of hippocampal neuronal ferroptosis, driven by excessive lipid peroxidation, has been demonstrated to ameliorate cognitive deficits (Sun et al., 2023). Studies utilizing DACD mouse models reveal a significant elevation in hippocampal levels of ferroptosis-mediated lipid peroxidation products and ROS, alongside a concomitant reduction in antioxidants such as GSH. The therapeutic agent liraglutide alleviates ferroptosis by attenuating lipid peroxidation, thereby mitigating its detrimental effects on hippocampal neurons and synaptic plasticity, and ultimately delaying the onset and progression of DACD (An et al., 2022). These findings underscore that the lipid metabolism network is a pivotal determinant of ferroptotic susceptibility (Figure 3). Cellular control over lipid peroxidation is achieved by modulating PUFA substrate availability through mechanisms encompassing β-oxidation, phospholipid remodeling, and lipid storage. Within the specific pathophysiology of diabetic cognitive dysfunction, a pathological triad comprising defective hypothalamic lipid sensing, disordered brain cholesterol metabolism, and high free fatty acid levels fosters a cerebral lipid milieu prone to ferroptosis. Therefore, therapeutic targeting of pivotal lipid metabolism enzymes, including ACSL4 and LPCAT3, or bolstering intrinsic antioxidant defenses, such as GPX4, through direct intervention in the core lipid peroxidation process, thus furnishing a critical theoretical foundation and promising intervention strategies for delaying or treating diabetes-associated cognitive decline.

### 2.3 Amino acid metabolism

#### 2.3.1 Xc-/GSH/GPX4 pathway

The cystine/glutamate antiporter (System Xc-) is a heterodimeric transporter composed of a light chain, xCT (SLC7A11), and a heavy chain, 4F2hc (SLC3A2), which are linked by a disulfide bridge. This system mediates the 1:1 exchange of extracellular cystine for intracellular glutamate. Following its import, cystine is reduced to cysteine, which serves as the rate-limiting precursor for the synthesis of GSH. As an essential cofactor for the antioxidant enzyme GPX4, GSH is critical for preventing lipid peroxidation; thus, direct inhibition of GPX4 or depletion of GSH can trigger the iron-dependent cell death pathway of ferroptosis (Wu et al., 2021). GPX4 catalyzes the oxidation of reduced GSH to oxidized glutathione (GSSG) while simultaneously reducing phospholipid hydroperoxides (PL-OOH) to their corresponding phospholipid alcohols (PL-OH) (Li L. et al., 2020; Jiang et al., 2021). Consequently, inhibition of GPX4 activity leads to the accumulation of lipid peroxides, thereby inducing ferroptosis. Agents such as erastin, glutamate, sorafenib, and sulfasalazine trigger this form of cell death by inhibiting the cystine/glutamate antiporter system Xc-, which reduces intracellular cystine availability, impairs glutathione biosynthesis, and ultimately suppresses GPX4 function (Xie et al., 2016; Wu et al., 2021; Yang et al., 2014). Within the diabetic milieu, characterized by chronic hyperglycemia and insulin resistance, the activity of System Xc- is inhibited. This suppression reduces cystine uptake, consequently depleting intracellular GSH and diminishing the activity of GPX4 (Duan et al., 2021). The compromised GPX4 function severely impairs the neuronal capacity to reduce phospholipid hydroperoxides, leading to the accumulation of lipid peroxides. Neurons in brain regions critical for cognitive functions, such as the hippocampus and cerebral cortex, exhibit heightened susceptibility to this oxidative damage and subsequent ferroptosis. Extensive loss or dysfunction of these neurons undermines synaptic plasticity and disrupts nerve conduction, which clinically manifests as impairments in learning, memory, executive function, and other cognitive domains (Wang D. et al., 2023).

#### 2.3.2 FSP1/CoQ/DHODH pathway

Ferroptosis suppressor protein 1 (FSP1) is recruited to the plasma membrane via N-terminal myristoylation, where it functions as an NAD(P)H-dependent oxidoreductase that catalyzes the reduction of coenzyme Q (CoQ) to its ubiquinol form (CoQH2). CoQ is a redox-active lipid essential for maintaining cellular homeostasis (Baschiera et al., 2021). The reduced form of CoQH2 functions as a potent, membrane-localized antioxidant that suppresses ferroptosis by inhibiting lipid peroxidation (Doll et al., 2019). Conversely, a depletion of the cellular CoQH2 pool can induce this form of iron-dependent cell death (Stockwell, 2022). Dihydroorotate dehydrogenase (DHODH), an enzyme located on the inner mitochondrial membrane, plays a dual role in pyrimidine biosynthesis and in the regeneration of CoQH2 through the reduction of CoQ. Pharmacological inhibition of DHODH may therefore promote ferroptosis by impairing CoQH2 production, which subsequently compromises the antioxidant activity dependent on this pathway (Wang X. et al., 2023; Jiang et al., 2023). FSP1 localized to the plasma membrane and DHODH within the mitochondrial matrix constitute two distinct cellular defense systems against ferroptosis, operating independently in separate subcellular compartments. The primary mechanism by which they suppress ferroptosis, specifically on the inner mitochondrial membrane, involves the reduction of CoQ10 to its antioxidant form, CoQ10H2 (Mao et al., 2021). Consequently, the development of pharmacological agents that inhibit FSP1 or modulate DHODH activity represents a promising therapeutic strategy for conditions such as DACD where ferroptosis is implicated.

#### 2.3.3 GCH1/BH4 pathway

GTP cyclohydrolase 1 (GCH1) serves as the rate-limiting enzyme for the synthesis of tetrahydrobiopterin (BH4), which acts as an essential cofactor for nitric oxide synthase (NOS); consequently, GCH1 activity indirectly regulates NOS function. BH4 is the dominant effector molecule within the GCH1/BH4 pathway. Genome-wide CRISPR activation screening has identified GCH1 and its metabolic product, BH4, as potent endogenous inhibitors of ferroptosis. The anti-ferroptotic activity of GCH1 is mediated primarily through two distinct mechanisms: firstly, by generating the lipophilic antioxidant BH4 (functionally analogous to coenzyme Q) to directly prevent lipid peroxidation; and secondly, by remodeling the lipid membrane environment, which involves increasing the levels of reduced CoQH2 and reducing the abundance of peroxidation-susceptible PUFA-containing phospholipids (Gao et al., 2022; Kraft et al., 2020). The Speedy/RINGO family protein Spy1 functions as a member of the cell cycle regulators that can inhibit neuronal ferroptosis through modulation of the GCH1/BH4 pathway, thereby attenuating lipid peroxidation (Wang Z. et al., 2023). Consequently, the GCH1/BH4 signaling axis plays a significant role in the pathogenesis of ferroptosis associated with cognitive decline.

#### 2.3.4 NRF2/SLC7A11/GPX4 pathway

Activation of SLC7A11 and GPX4 expression via nuclear factor erythroid 2-related factor 2 (NRF2) is critical for ferroptosis inhibition (Liu et al., 2023; Zhang et al., 2024). NRF2 functions as a central transcriptional regulator that induces cytoprotective genes such as SLC7A11 and GPX4, thereby counteracting oxidative stress in cognitive impairment (Dodson et al., 2019; El-Gohary et al., 2024; Lane et al., 2023). SLC7A11 functions as a transmembrane cystine/glutamate antiporter responsible for the cellular uptake of cystine, which is essential for glutathione biosynthesis (Berndt et al., 2024). GPX4 is a selenocysteine-containing enzyme that mitigates lipid peroxidation by reducing phospholipid hydroperoxides to their corresponding alcohols, thereby suppressing ferroptosis (Gleason and Bush, 2021). The compound ML385 acts as an inhibitor of NRF2 by binding to its Neh1 domain, thereby blocking heterodimerization with Maf proteins and subsequent transcription of antioxidant response element-dependent genes (Yang et al., 2024). One investigation demonstrated that in an animal model of cognitive dysfunction, administration of Ganoderic Acid A (GAA) resulted in a marked enhancement in the protein levels of NRF2, SLC7A11 and GPX4. This GAA-induced upregulation was significantly antagonized by co-treatment with the NRF2 inhibitor ML385 (Lu et al., 2025). Activation of the NRF2/GPX4 signaling axis has been established as a mechanism that suppresses ferroptosis, consequently decelerating the progression of DACD. Targeting the ferroptotic process, a key pathophysiological event verified in a db/db mouse model of DACD, through the administration of dendrobine was observed to attenuate neuronal damage. This protective effect was associated with the elevated protein levels of NRF2 and GPX4 (Shi et al., 2023). Therefore, a strategic approach to mitigate cognitive decline involves the regulation of the integrated NRF2/SLC7A11/GPX4 pathway to counteract lipid peroxidation and ferroptotic cell death.

#### 2.3.5 AMPK pathway

**FIGURE 4:**
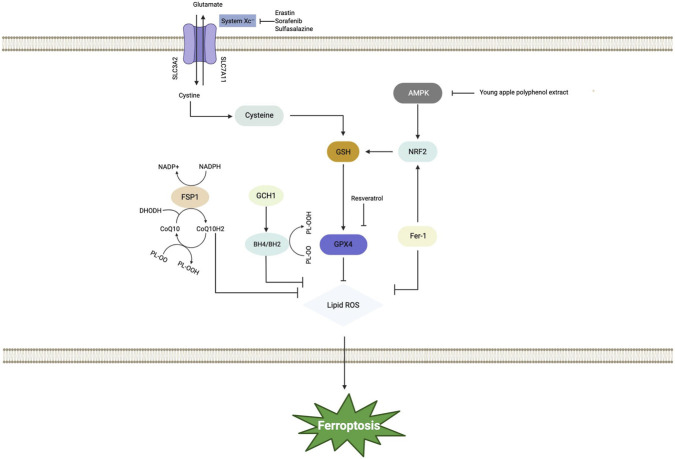
The main molecular mechanisms of ferroptosis-related diabetes-associated cognitive dysfunction include amino acid metabolism. Centered on the System Xc-GSH-GPX4 axis and supported by auxiliary defense pathways such as FSP1-CoQ10 and GCH1-BH4, cells cooperatively eliminate Lipid ROS. Various inducers (e.g., Erastin, Sorafenib) disrupt redox homeostasis by inhibiting System Xc^−^ or GPX4, whereas inhibitors (e.g., Ferrostatin-1, apple polyphenol extract) enhance cytoprotection through direct antioxidant effects or by activating the AMPK/NRF2 signaling pathway. Ultimately, these integrated pathways regulate the level of lipid peroxidation, determining whether a cell undergoes survival or ferroptosis. Abbreviations: AMPK-AMP, activated protein kinase; BH2, Dihydrobiopterin; BH4, Tetrahydrobiopterin; CoQ10, Coenzyme Q10; CoQ10H2, Ubiquinol; DHPDH, Dihydropyrimidine Dehydrogenase; Fer-1, Ferrostatin-1; FSP1, Ferroptosis Suppressor Protein 1; FTH1, Ferritin Heavy Chain 1; FTL, Ferritin Light Chain; GCH1-GTP Cyclohydrolase 1; GPX4, Glutathione Peroxidase4; GSH, Glutathione; NADP + , Nicotinamide Adenine Dinucleotide Phosphate; NADPH, Reduced Nicotinamide Adenine Dinucleotide Phosphate; NRF2, Nuclear Factor Erythroid2- Related Factor 2; PL-00, Phospholipid Peroxyl Radical; PL-OOH- Phospholipid Hydroperoxide; SLC3A2, Solute Carrier Family 3 Member 2; SLC7A11, Solute Carrier Family 7 Member 11.

AMP-activated protein kinase (AMPK) is a heterotrimeric serine/threonine kinase complex comprising α(catalytic), β (scaffold), and γ(regulatory) subunits. It functions as a master regulator of cellular energy homeostasis. AMPK activation has been demonstrated to suppress ferroptosis through mediating the phosphorylation of acetyl-CoA carboxylase (ACC) and regulating the biosynthesis of PUFAs (Lee et al., 2020). Inflammatory stimuli and elevated levels of ROS can modulate the AMPK signaling pathway, thereby promoting the onset of ferroptosis (Chen et al., 2023). This relationship is corroborated by evidence from a type 2 diabetes-associated cognitive dysfunction mouse model, in which ferroptosis mediated by the AMPK pathway was significantly enhanced (Guo et al., 2023). Activation of AMPK has been shown to ameliorate cognitive impairment in DACD via the downregulation of key ferroptosis-related proteins, including ferritin, GPX4, and SLC7A11 in hippocampal neurons (Xie et al., 2023). Consequently, further investigation into the ferroptosis-mediated AMPK pathway holds promise for identifying novel therapeutic targets for DACD. In summary, amino acid metabolism precisely regulates the ferroptotic process through several distinct pathways, such as the Xc-/GSH/GPX4, FSP1/DHODH/CoQ, GCH1/BH4, NRF2/SLC7A11/GPX4, and AMPK (Figure 4). Multiple interconnected pathways play a crucial role in maintaining neuronal redox homeostasis by facilitating glutathione synthesis, offering auxiliary antioxidant protection, activating comprehensive stress defense systems, and coupling with energy metabolism. In diabetic cognitive dysfunction, metabolic disturbances such as hyperglycemia can concurrently impair the function of these pathways, thereby increasing neuronal susceptibility to ferroptosis and resulting in damage to critical brain regions like the hippocampus. A targeted combinatorial approach, for instance, simultaneously enhancing GPX4 activity and modulating AMPK signaling, presents a promising therapeutic strategy for intervening in the disease process.

## 3 Application of ferroptosis inhibitors in diabetes-associated cognitive dysfunction

Although inhibitors of ferroptosis demonstrate efficacy in ameliorating DACD, no specific therapeutic agent has yet received clinical approval for this indication. Given the established role of ferroptosis in DACD pathogenesis, key regulatory molecules within this cell death pathway are considered promising novel therapeutic targets. Several compounds targeting ferroptosis have shown beneficial effects in preclinical models of DACD (Table 2).

**TABLE 2:** Summary of ferroptosis inhibitors for Diabetes-associated cognitive dysfunction.

Drug/Compound	Models	Mechanism	Key experimental outcome/Effect	References
Ferrostatin-1	Aβ-treated primary neurons; Aβ-injected mouse hippocampus	Prevents lipid peroxidation	Reduced neuronal death *in vitro*; improved spatial memory (shortened escape latency in Morris water maze) *in vivo*	Bao et al. (2021)
Deferoxamine	T2DM male rats (post-stroke); Brain microvascular endothelial cells from diabetic GK rats	Lowers the expression of ferroptosis markers (IREB2 and ACSL4) in endothelial cells	Prevents post-stroke vasoregression and microglia activation; improves BBB integrity and functional outcomes in diabetic rats; inhibits ferroptosis *in vitro*	Abdul et al. (2021)
Intranasal deferoxamine	STZ-induced diabetic rat model	Chelates iron; reduces oxidative stress; restores insulin receptor expression	Significantly improves spatial memory (shorter escape latency in Morris water maze)	Fine et al. (2017)
Phytic acid	Patients with T2DM and MCI	Iron chelation; inhibition of advanced glycation end product formation	Improve cognitive function and attenuate the progression from MCI to dementia (This is an ongoing clinical trial; results are pending)	Pujol et al. (2024)
Liraglutide	db/db mice	Attenuates oxidative stress and iron overload; downregulates TfR1, upregulates FPN1/FTH	Improves spatial memory; reduces escape latency and increases platform crossings in Morris water maze	An et al. (2022)
Empagliflozin	Neuronal OGD/R model	Activates the Nrf2/HO-1 antioxidant signaling pathway	Alleviates oxidative stress and restores cellular iron homeostasis	Ma et al. (2025)
Metformin	Cellular/Osteoarthritis models	Modulates the P53/SLC7A11 axis	Suppresses cellular ferroptosis; alleviates metabolic imbalance	Jiaxin et al. (2025)
Erythropoietin	PC12 cells + STZ/HFD mice	Reduces brain iron overload and lipid peroxidation; upregulates GPX4/ferritin, downregulates ACSL4	Ameliorates cognitive deficits; shortens escape latency in Morris water maze	Guo et al. (2023)
Young apple polyphenol extract	STZ/HFD mice	Improves mitochondrial TCA cycle homeostasis; promotes AMPK phosphorylation; reduces ROS	Suppresses neuronal lipid peroxidation; improves cognitive outcomes	Fang et al. (2025)
Resveratrol	HT22 cells + db/db mice	Activates PKA; regulates expression of GPX4 and SLC7A11	Inhibits ferroptosis; offers neuroprotection	Cao et al. (2023)

### 3.1 Ferrostatin-1

Ferrostatin-1 (Fer-1) is a potent and selective inhibitor of ferroptosis. Its mechanism of action involves directly neutralizing lipid radicals and reducing the intracellular labile iron pool, thereby attenuating the iron-catalyzed Fenton reaction at its source and diminishing subsequent ROS generation. Experimental evidence demonstrates that Fer-1 inhibits ferroptosis by reducing ROS levels and modulating key regulatory proteins including NRF2 and GPX4, which is associated with improved cognitive function in model systems. Specifically, Fer-1 treatment reduced neuronal death by approximately 50% (p < 0.01) and significantly enhanced memory performance in mice, evidenced by a ∼50% increase in target quadrant dwell time during the Morris water maze probe trial (p < 0.05) (Bao et al., 2021). However, clinical trials investigating Fer-1 for the treatment of DACD are currently absent; consequently, its therapeutic efficacy and potential adverse effects in DACD necessitate further evaluation.

### 3.2 Iron chelators

The iron chelator deferoxamine (DFO) exerts multi-targeted inhibitory effects on ferroptosis in the context of DACD. Its mechanism involves high-affinity binding to Fe^3+^ to form stable ferrioxamine complexes, which are subsequently eliminated from the body. By sequestering free iron, DFO prevents iron-catalyzed lipid peroxidation-the hallmark of ferroptosis. Experimental evidence from diabetic rat models indicates that DFO treatment significantly downregulates cerebral expression of key pro-ferroptotic proteins, iron-responsive element-binding protein 2 (IREB2) and ACSL4, consequently suppressing ferroptosis (Abdul et al., 2021; Chen, 2026). However, the clinical application of DFO is limited by its pharmacokinetic properties: a short plasma half-life, reliance on prolonged intravenous or intramuscular infusion for conventional administration, and difficulty in achieving effective therapeutic concentrations in the brain parenchyma due to the presence of the BBB (Kosyakovsky et al., 2021). The intranasal delivery strategy offers a breakthrough for this challenge. This route utilizes the olfactory and trigeminal nerve pathways to directly transport drugs extracellularly to the central nervous system, bypassing the BBB and enabling rapid brain-targeted delivery (Rao et al., 2025). Studies have shown that Intranasal DFO demonstrates significant cognitive benefits across both pathological and healthy models. In a streptozotocin-induced diabetic rat model, DFO treatment significantly improved spatial memory, evidenced by shorter escape latencies in the Morris Water Maze (p < 0.05). This was coupled with a reduction in oxidative stress (p < 0.05) and restored insulin receptor expression (p < 0.05) (Fine et al., 2017). Remarkably, DFO also enhanced working memory in healthy C57 mice. Treated animals showed significantly fewer errors and shorter latencies in the Radial Arm Water Maze (p < 0.05) (Fine et al., 2020). These findings suggest its neuroprotective effects are partially independent of the diabetic pathological state.

Phytic acid (phytate), a naturally occurring potent chelator of metal ions, can sequester iron ions and reduce iron-dependent lipid peroxidation, thereby exhibiting the potential to inhibit ferroptosis. This hypothesis is under clinical investigation via the PHYND trial protocol, which aims to assess whether oral phytic acid supplementation can ameliorate cognitive decline in patients with T2DM and mild cognitive impairment (Pujol et al., 2024). A positive outcome from this trial would support its therapeutic potential. Collectively, these findings indicate that iron chelators can effectively suppress the ferroptotic process through multiple pathways in DACD. Among them, DFO demonstrates considerable therapeutic potential due to its multi-target mode of action, including iron chelation, inhibition of iron-catalyzed lipid peroxidation, and regulation of key proteins. The intranasal administration method further enhances brain targeting and reduces systemic exposure risks, presenting a promising intervention strategy for DACD.

### 3.3 Anti-diabetic drugs

Emerging evidence indicates that, beyond glucoregulatory actions, certain anti-diabetic agents can ameliorate DACD by interfering with the ferroptosis pathway. Specifically, glucagon-like peptide-1 receptor agonists (GLP-1RAs; e.g., liraglutide) suppress ferroptosis by attenuating oxidative stress and iron overload, thereby preserving cognitive function in experimental models of diabetes. Treatment (200 μg/kg/day, 5 weeks) improved spatial memory in db/db mice, reducing Morris water maze escape latency (p < 0.001) and increasing platform crossings (p < 0.01). Mechanistically, it attenuated oxidative stress by decreasing ROS/MDA and enhancing superoxide dismutase/glutathione peroxidase (SOD/GSH-Px) activity (p < 0.05), while rebalancing iron homeostasis through TfR1 downregulation and FPN1/FTH upregulation (p < 0.05) (An et al., 2022). Evidence suggests that sodium-glucose cotransporter 2 (SGLT2) inhibitors, such as empagliflozin, alleviate oxidative stress and restore cellular iron homeostasis by activating the Nrf2-associated antioxidant signaling pathway, thereby effectively inhibiting neuronal ferroptosis (Ma et al., 2025). Separately, metformin has been shown to suppress cellular ferroptosis through modulation of the P53/SLC7A11 axis (Jiaxin et al., 2025).

In addition to the aforementioned pharmacological agents, drug delivery strategies that directly target the central nervous system have shown promising application prospects. For instance, a randomized, double-blind, placebo-controlled phase 2 clinical trial demonstrated that intranasal insulin treatment improved walking speed, cerebral blood flow, executive function, and verbal memory in patients with type 2 diabetes (Novak et al., 2022), suggesting its potential for cognitive enhancement. Nonetheless, direct experimental evidence regarding the regulation of ferroptosis by medications such as empagliflozin and metformin in the context of DACD remains relatively limited, particularly lacking validation of the underlying mechanisms in animal models of DACD. Therefore, future studies are needed to further elucidate the specific molecular pathways through which these agents ameliorate DACD via the ferroptosis pathway, both *in vivo* and *in vitro*, so as to provide a solid foundation for clinical therapeutic strategies.

### 3.4 Erythropoietin

The glycoprotein hormone erythropoietin (EPO), comprising 165 amino acids, demonstrates significant multifunctional neuroprotective properties. In experimental models of T2DM, EPO administration ameliorates cognitive deficits by mitigating cerebral iron overload, suppressing the ferroptosis pathway, and modulating the expression of key regulatory proteins such as GPX4 and ferritin. In the Morris water maze test, EPO-treated mice exhibited markedly shortened escape latencies compared to the diabetic model group (p < 0.01). This cognitive improvement was mechanistically linked to the alleviation of cerebral iron overload—cortical iron content was significantly reduced following EPO treatment (p < 0.05). Furthermore, EPO effectively suppressed ferroptosis by decreasing lipid peroxidation, indicated by reduced MDA levels both *in vivo* (p < 0.05) and *in vitro*, and by modulating key regulatory proteins: it up-regulated the expression of GPX4 and ferritin (FTH1 and FTL) (p < 0.05), while down-regulating the ferroptosis promoter ACSL4 (Guo et al., 2023). Future investigations should prioritize elucidating the upstream signaling mechanisms through which EPO exerts its anti-ferroptotic effects.

### 3.5 Young apple polyphenol extract

Young apple polyphenol extract (YAPE) is a polyphenol-rich concentrate obtained from thinned immature apples, with chlorogenic acid, phlorizin, and hyperoside identified as its principal bioactive constituents. Research demonstrates that YAPE ameliorates DACD by reducing ROS levels through a dual action: enhancing mitochondrial tricarboxylic acid (TCA) cycle homeostasis and promoting AMPK phosphorylation. This coordinated mechanism effectively suppresses neuronal lipid peroxidation, thereby improving cognitive outcomes (Fang et al., 2025).

### 3.6 Resveratrol

Resveratrol, a naturally occurring non-flavonoid polyphenolic stilbenoid found in plants such as grapes, peanuts, and mulberries, downregulates the expression and activity of phosphodiesterase 4D (PDE4D). This inhibition elevates intracellular cyclic adenosine monophosphate (cAMP) levels, thereby potentiating protein kinase A (PKA) signaling. The activation of PKA subsequently modulates the expression of key ferroptosis regulators, including GPX4 and SLC7A11, leading to the suppression of ferroptosis (Cao et al., 2023). Furthermore, resveratrol enhances mitochondrial function via this PKA-dependent pathway, which may confer indirect protection against ferroptosis, offering a novel therapeutic perspective for DACD.

## 4 Challenges, limitations, and future directions

While evidence linking ferroptosis to the pathogenesis of DACD is compelling and continues to accumulate, this area of research remains in a relatively early stage. The translation of these promising preclinical findings into viable clinical applications faces considerable challenges. This section examines current limitations and suggests constructive directions for future research.

### 4.1 The translational gap between preclinical models and human disease

Current insights into the role of ferroptosis in DACD are largely based on evidence from preclinical models, including genetically modified or chemically induced rodents (e.g., db/db mice, STZ-induced rats) and immortalized cell lines (e.g., PC12 cells) (An et al., 2022; Guo et al., 2023). Although indispensable for elucidating pathogenic mechanisms, these models cannot fully replicate the intricate, multifactorial nature of human DACD—a condition that progresses recent decades within a context of aging, genetic heterogeneity, and comorbid systemic diseases. Fundamental species differences in iron metabolism, antioxidant defense systems, and neural architecture challenge the direct extrapolation of preclinical findings to humans. Therefore, future studies should prioritize the development and application of human-relevant models. These include induced pluripotent stem cell (iPSC)-derived neuronal and glial cultures from diabetic patients with or without cognitive impairment, which will better model human-specific pathophysiology and facilitate the validation of therapeutic targets identified in preclinical studies (Gleason and Bush, 2021).

### 4.2 Hurdles in the clinical development of ferroptosis inhibitors

Ferroptosis inhibitors, exemplified by Fer-1, have proven effective in preclinical studies; however, their path from the laboratory to the clinic encounters notable hurdles (Bao et al., 2021). Key challenges stem from their unfavorable pharmacokinetic properties, notably metabolic instability and limited bioavailability, which thereby restrict their therapeutic utility. The BBB poses a fundamental challenge by restricting the CNS delivery of most systemically circulating drugs. Furthermore, the risks of off-target effects and the long-term toxicity arising from sustained inhibition of a physiological pathway require careful evaluation. Consequently, advancing this field will require a concerted focus on either designing new ferroptosis inhibitors with optimized CNS penetration or engineering delivery systems that circumvent the BBB. The intranasal route, for instance, has demonstrated considerable potential in preclinical models, such as with intranasal DFO, for achieving direct brain delivery (Fine et al., 2017; Kosyakovsky et al., 2021).

### 4.3 The critical need for biomarkers of brain ferroptosis *in vivo*

The advancement of ferroptosis-targeted therapies in clinical trials is hampered by a critical bottleneck: the absence of validated, non-invasive biomarkers to directly detect and monitor ferroptosis within the brains of living patients. Current imaging biomarkers like QSM provide only indirect evidence by mapping iron deposition, a necessary but insufficient condition for ferroptosis. For instance, observed iron accumulation in the basal ganglia of diabetic patients signals risk, not confirmation, of active cell death (Yang et al., 2018; Cheng et al., 2025). It is therefore imperative that future biomarker discovery efforts target molecular events that are directly specific to ferroptosis. Promising candidates include specific lipid peroxidation products (e.g., oxidized phosphatidylethanolamine species) in cerebrospinal fluid, or novel imaging ligands for key targets such as GPX4 (Kagan et al., 2017). Establishing a predictive value for such biomarkers against cognitive decline will be crucial for stratifying patients who are most likely to respond to anti-ferroptotic therapies.

### 4.4 Mechanistic exploration of repurposed anti-diabetic drugs

Preclinical data reporting the neuroprotective effects of drugs such as GLP-1 receptor agonists (e.g., Liraglutide) and SGLT2 inhibitors raise the possibility that their benefits extend beyond glycemic control to potentially encompass the inhibition of ferroptosis (An et al., 2022; Ma et al., 2025). However, the exact mechanisms by which these drugs influence ferroptosis pathways in the brain are still incompletely understood and require direct experimental confirmation. A key objective is to determine whether these benefits are mediated by direct modulation of core ferroptosis components (e.g., GPX4 or SLC7A11) or secondary to systemic metabolic improvements (Xie et al., 2023). Consequently, elucidating these precise molecular mechanisms will not only substantiate their therapeutic repurposing but also pave the way for developing more targeted, CNS-specific regimens.

### 4.5 Designing future clinical trials

The design of future clinical trials targeting ferroptosis in DACD must be thoughtful and evidence-based. Key considerations include: Patient selection. The trial enrolled well-characterized T2DM patients presenting with early-stage, objectively verified cognitive impairment. Study participants were stratified based on key risk factors, including ApoEε4 genotype status and evidence of cerebral iron overload as quantified by QSM (Irie et al., 2008; Cheng et al., 2025). Intervention Strategies. A promising path is the strategic repurposing of compounds with proven safety profiles. This includes investigating iron chelators or determining the CNS-effective doses of GLP-1 RAs to rapidly evaluate their anti-ferroptotic efficacy (Fine et al., 2017; An et al., 2022). Combination therapies concurrently targeting distinct nodes within the ferroptosis pathway (e.g., pairing an iron chelator with a lipid antioxidant) represent a promising strategy for achieving synergistic anti-ferroptotic efficacy. Outcome Measures. A comprehensive assessment strategy should be implemented, co-primary endpoints that integrate sensitive, multi-domain cognitive tests with quantifiable biomarkers of central ferroptosis. This approach is essential to objectively demonstrate target engagement and establish a conclusive link to clinical efficacy (Dodson et al., 2019).

In conclusion, while significant progress has been made in elucidating the role of ferroptosis in DACD, translating these mechanistic insights into effective therapies remains a formidable challenge. Addressing these challenges through collaborative efforts spanning basic science, translational neurobiology, and clinical neurology will be crucial for realizing the potential of targeting ferroptosis to alleviate the burden of DACD.
